# A theoretical model of secular body mass index dynamics among Russian adults under changing socio-economic conditions

**DOI:** 10.1186/s40101-025-00415-5

**Published:** 2025-12-29

**Authors:** Marina A. Negasheva, Olga A. Kuznetsova, Ainur A. Khafizova, Alla A. Movsesian

**Affiliations:** Department of Anthropology, Lomonosov State University, Moscow, Russia

**Keywords:** Secular trend, Socio-economic factors, Body mass index, Regression modeling, Russia

## Abstract

**Background:**

Body mass index (BMI) is a key indicator of population health and often shifts alongside socio-economic change. Few studies have tracked these dynamics over long periods in transitional economies. Russia’s late-twentieth-century transformations offer a rare opportunity to examine such links. This study develops and validates a time-series model of BMI in 19 years old, relating changes in socio-economic and demographic indicators to BMI trends and producing scenario-based forecasts.

**Materials and methods:**

We analyzed national time-series data published by the NCD Risk Factor Collaboration for 1975–2016 (males and females, age 19), along with indicators of urbanization, fertility, infant and all-cause mortality, life expectancy, and nutritional proxies (protein supply, animal-source calories, meat). Predictors were standardized. Per sex, we estimated the following: (i) first-difference OLS, (ii) dynamic regressions with a lagged BMI term (ARDL(1,0)), and (iii) smooth-trend models with a natural cubic spline in year. Diagnostics included augmented Dickey–Fuller and Durbin–Watson tests. Model selection triangulated elastic net, partial least squares, and stepwise regression. Rolling-origin one-step-ahead forecasts used only information available at time t; bootstrap resampling assessed sign stability.

**Results:**

Across specifications, the urbanization share was the most robust correlate of BMI. For males, higher urbanization was consistently and inversely associated with BMI; for females, the association was small and model sensitive (frequently negative but not uniformly significant). Effects of life expectancy and mortality attenuated and often lost significance once smooth time structure was included, indicating shared long-run movement rather than distinct short-run covariation; infant mortality added little independent signal. Nutrition proxies contributed limited, non-robust information. ARDL(1,0) one-step-ahead forecasts outperformed random-walk and trend-only baselines. Under a baseline scenario (continuation of recent socio-economic patterns), projected BMI in 2050 is approximately 26.6 kg/m^2^ (males) and 26.7 kg/m^2^ (females). Forecasts use only information available at time t (lagged predictors/nowcasts) and are conditional on assumed exogenous trajectories; longer-horizon projections are scenario based rather than unconditional.

**Conclusions:**

After explicit treatment of nonstationarity, macrodemographic structure, especially urbanization, shows the most consistent links to BMI at age 19, whereas national-scale nutrition proxies are weak at this grain. Findings are descriptive, not causal; forecasts should be interpreted with caution. Incorporating finer-grained behavioral, dietary, and environmental data will help clarify mechanisms and improve long-term forecasting.

**Supplementary Information:**

The online version contains supplementary material available at 10.1186/s40101-025-00415-5.

## Background

One of the central areas of research in biological anthropology is the study of variation in the morpho-functional status of humans across time and space, as well as the identification of key environmental factors that influence this variability. In the context of the modern post-industrial, information-driven society at the turn of the twentieth and twenty-first centuries, particular attention has been paid to the socio-economic factors that affect intergenerational changes in overall body size. This includes both global trends and regional specificities. Body mass index (BMI) is among the most widely used indicators in biological anthropology and medicine for assessing total body size and relative body mass [1, 2]. Because secular changes in BMI reflect shifts in growth, nutrition, and environment, tracking these trends and their determinants is central to understanding microevolutionary processes in contemporary human populations. Although BMI is an imperfect proxy for adiposity—elevated values can also reflect greater muscle mass or population-specific fat distribution [3–5]—it remains the most practical standard metric for large-scale analyses of secular trends, owing to its ease of calculation and broad data availability.

Over the past century, substantial shifts have been observed in key morphological traits—particularly height and body mass—across many regions of the world [6–8]. In Europe, for example, notable gains in height and average body mass have been especially prominent over the past three decades [9–12]. A similar pattern has been documented in the Chinese population (Ji and Chen, 2008). A central challenge in contemporary human biology is to identify the factors driving these morphological changes and to understand how they interact with broader environmental and social conditions [13]. Socio-economic status, dietary habits, and environmental conditions are among the primary external determinants thought to influence variation in BMI [14, 15]. Another important factor is the decline in physical activity: with technological advancement and increased automation, activity levels have fallen, likely contributing to the rising prevalence of overweight and obesity [16]. There is also evidence that even minimal physical activity can effectively reduce BMI [17].

Since the second half of the twentieth century, intergenerational increases in BMI have been documented globally among both males and females across all age groups [18–20]. A comprehensive analysis of secular trends in adult BMI, based on data from more than 19.2 million individuals across 186 countries, revealed that between 1975 and 2014, average BMI rose from 21.7 kg/m^2^ to 24.2 kg/m^2^ in males—at an average rate of 0.63 kg/m^2^ per decade—and from 22.1 kg/m^2^ to 24.4 kg/m^2^ in females, increasing by approximately 0.59 kg/m^2^ per decade [18]. In most countries, national-level BMI trajectories closely align with global trends. Notable increases in BMI have been observed among youth in Germany [21], the Netherlands [22], and Poland [23, 24]. In Russia, secular increases in body weight, BMI, and indicators of fat accumulation have also been reported across various population groups [25–27].

These increases in average body weight have been accompanied by rising rates of overweight and obesity in many countries [18, 20]. However, recent studies suggest a potential shift in these trends among younger generations. In particular, some findings point to a decline in the prevalence of overweight and obesity—based on BMI assessments—among children and adolescents [28, 29]. In some countries, for example, in Japan, an increase in the proportion of underweight females has also been reported [30].

Despite extensive documentation of BMI trends, few studies have developed formal models that link socio-economic dynamics to long-term BMI trajectories, particularly in the Russian context. Most existing research does not identify the most influential drivers of change or offer forecasts of future trends in body composition. As a result, there remains no established framework that quantitatively relates time-varying socio-economic and demographic factors to intergenerational (diachronic) changes in somatic traits or that projects future trajectories in body size under plausible shifts in external conditions.

This gap is especially striking in Russia, where BMI dynamics have unfolded alongside profound political, economic, and demographic transformations. Despite rich datasets and growing public health concerns, the long-term interaction between social change and population-level morphology remains underexplored. The need for such theoretical syntheses has grown and has become feasible with the accumulation of large, multi-indicator datasets [31]. The availability of open-source, aggregated time series, where local fluctuations are partially averaged out, enables clearer identification of long-term temporal patterns.

The present study addresses this problem by developing and testing a forecasting model of adult BMI in Russia, using historical time series of socio-economic indicators. Specifically, we apply a combination of time-series modeling, regularization, and validation techniques to identify robust predictors and forecast future dynamics in mean BMI.

Beyond its technical value, such modeling has both applied and theoretical significance. Anticipating BMI trajectories can inform public health policy, resource allocation, and preventive strategies for obesity-related conditions. From a broader anthropological perspective, modeling these regularities contributes to our understanding of microevolutionary patterns in somatic variation across contemporary human populations.

## Materials and methods

This study draws on average BMI data for 19-year-old males and females in Russia from 1975 to 2016, as reported by the NCD Risk Factor Collaboration (NCD-RisC, 2017).

This specific age group was selected for several reasons. First, by age 19, growth processes are largely complete in both sexes, and individuals have generally reached their definitive somatic status—meaning that key morphological traits have stabilized. At the same time, involutional (age-related degenerative) changes have not yet begun. Moreover, the prevalence of age-dependent diseases and occupational morphological distortions remains very low at this age. Thus, 19 years old represent an optimal “model” age group for analyzing intersystem relationships in a conditionally healthy population that has attained definitive somatic status and is situated at the most stable stage of ontogenetic development in terms of morphofunctional characteristics.

To evaluate the influence of socio-economic and demographic factors on secular trends in BMI and to construct a theoretical model linking BMI to long-term changes in these variables, a comprehensive set of indicators was analyzed. These included both socio-economic and nutritional parameters: average monthly per capita income in rubles, the Gini index (World Bank), total urban population and its share of the overall population, life expectancy at birth, urban birth rate, total fertility rate, death rate, infant mortality, daily protein supply per capita (g), daily caloric intake from animal protein (per person), animal-source protein consumption per capita (g), per-capita meat supply in Russia (kg/year), and total meat consumption in Russia.

### Statistical analysis

All statistical analyses were carried out in R (v4.3), using a combination of base functions and specialized packages. Before modeling, all socio-economic and demographic variables were standardized with the scale() function so that each was expressed as a z-score—centered on its mean and scaled by its standard deviation. This transformation makes regression coefficients directly comparable across predictors. Table 1 illustrates the effect of standardization by showing raw values alongside standardized values; entries are reported as raw (z score) for each variable.

**Table 1:** Comparison between standardized (z-score) values and original raw values

Year	Line	Urban pop (millions)	Life exp. (years)	Urban birth rate (per 1000)	Urban fertility rate (births/woman)	Death rate (per 1000)	Infant mortality (per 1000)	Protein supply (g/day)	Animal calories/day (kcal/person)	Meat supply (kg/year)	BMI male (kg/m^2^)	BMI female (kg/m^2^)
1975	Raw	88.9	67.7	16.1	2.0	9.8	24.8	106.5	211.4	60.0	24.3	26.0
1975	z	−2.9	−0.1	1.2	1.1	−1.6	1.7	1.0	0.5	−0.3	−1.5	−2.3
2000	Raw	107.4	65.5	8.7	1.2	15.3	15.5	85.2	165.5	45.0	25.1	26.6
2000	z	0.5	−1.1	−1.3	−1.5	1.1	0.2	−1.9	−1.9	−1.9	0.1	0.5
2016	Raw	108.6	71.7	12.9	1.8	12.9	6.1	101.4	214.0	74.0	26.0	26.7
2016	z	0.7	1.6	0.1	0.4	−0.1	−1.4	0.3	0.6	1.2	1.9	0.8

Line “Raw” gives original values; line “z” gives standardized values (z-scores)

A multistep procedure was then applied to select the most relevant predictors for the model. As a first exploratory step, we examined the strength of association between each potential predictor and BMI by calculating Pearson correlation coefficients with psych::corr.test() [32]. This allowed us to identify variables that were strongly related to BMI and to flag those with negligible or redundant correlations.

Because the dataset spans more than four decades, many series exhibit clear long-term trends. We first screened each series with the augmented Dickey–Fuller (ADF) test (tseries::adf.test()) to assess stationarity. We then attempted automated transformations using healthyR.ts::auto_stationarize(). For several variables—including urban birth rate, urban fertility rate, infant mortality, and male BMI—these procedures did not consistently yield stationary series, likely because their nonstationarity reflects structural breaks as well as deterministic trends. As not all variables could be made stationary (including the key outcome, male BMI), and detrending would eliminate substantively important long-run dynamics, we retained the original level series without modification in those specifications where preserving trend information was essential.

Given the prevalence of nonstationarity and the risk of trend-driven spurious correlation, we estimated three complementary specifications for each sex to ensure robustness to modeling choices: (i) *first-difference OLS *(with Newey–West SEs), (ii) a *dynamic ARDL(1,0)* model with lagged BMI, and (iii) a *smooth-trend* model that includes a natural cubic spline in calendar year.

#### First-difference model

Year-to-year changes in BMI were regressed on corresponding changes in each predictor:

$$\Delta{\mathrm{BMI}}_{\mathrm t}\:=\:{\mathrm\alpha}_{\mathrm s}\:+\:{\mathrm\Sigma}_{\mathrm j}\;{\mathrm\beta}_{(\mathrm j,\mathrm s)}\;\mathrm\Delta\;{\mathrm X}_{(\mathrm j,\mathrm s,\mathrm t)}\:+\:{\mathrm\varepsilon}_{\boldsymbol(\mathrm s,\mathrm t)}.$$

This specification controls for shared level trends, focusing on short-term co-movements; Newey–West standard errors (sandwich::NeweyWest()) with a 1-year lag were applied to correct for autocorrelation and heteroskedasticity.

#### Dynamic model (ARDL(1,0))

BMI was regressed on its own 1-year lag and the contemporaneous value of each predictor:

$${\mathrm{BMI}}_{\mathrm t}\:=\:{\mathrm c}_{\mathrm s}\:+\:{\mathrm\varphi}_{\mathrm s}\;{\mathrm{BMI}}_{(\mathrm t-1)}\:+\:{\mathrm\Sigma}_{\mathrm j}\;{\mathrm\gamma}_{(\mathrm j,\mathrm s)}\;{\mathrm X}_{(\mathrm j,\mathrm s,\mathrm t)}\:+\:{\mathrm\varepsilon}_{(\mathrm s,\mathrm t)}.$$

This structure explicitly captures inertia in BMI while estimating immediate effects of the socio-economic variables.

#### Smooth-trend model

BMI was modeled as a function of the predictors plus a natural cubic spline in calendar year with four degrees of freedom (splines::ns()):

$${\mathrm{BMI}}_{\mathrm t}\:=\:{\mathrm a}_{\mathrm s}\:+\:{\mathrm\Sigma}_{\mathrm j}\;{\mathrm\delta}_{(\mathrm j,\mathrm s)}\;{\mathrm X}_{(\mathrm j,\mathrm s,\mathrm t)}\:+\:\mathrm{ns}\;(\mathrm t,\mathrm{df}\:=\:4)\:+\:{\mathrm\varepsilon}_{(\mathrm s,\mathrm t)}.$$

This absorbs gradual common trends across variables while isolating more specific associations with BMI.

For each model, we computed Durbin–Watson statistics (lmtest::dwtest()) and reapplied the ADF test to the residuals to confirm that autocorrelation had been reduced and that residuals were stationary (Supplementary Tables S2 and S3).

To evaluate predictive performance, we used a rolling-origin (walk-forward) validation approach. At each step, the model was re-estimated on data available up to year t and used to forecast BMI for year t + 1, using only information available at time t (lagged predictors; where contemporaneous values were required, simple nowcasts such as random walk/AR(1) were used).

The ARDL(1,0) model’s forecasts were compared against two baselines: a naïve random walk (projecting next year’s BMI as equal to the current value) and a quadratic time-trend model (BMI~year + year^2^). Forecast accuracy was assessed using mean absolute error (MAE), root-mean-squared error (RMSE), and mean absolute percentage error (MAPE). Across both sexes, the ARDL model consistently outperformed the baselines (Table S4).

Given the relatively small sample size (*N* = 42 years), we also examined the stability of the ARDL coefficients using a moving-block bootstrap with a block length of 3 years and 500 replications. For each predictor, we report percentile-based 95% confidence intervals and the proportion of bootstrap samples in which the sign of the coefficient was preserved (Table S5). Life expectancy and the share of the urban population emerged as the most robust predictors, showing high sign stability across bootstrap runs.

Subsequently, *elastic net regression* was performed to identify key predictors and reduce model complexity. This method combines two types of regularization penalties—L1 (Lasso) and L2 (Ridge)—to shrink coefficient magnitudes and select variables with the strongest predictive power [33, 34]. Lasso regression (L1 regularization) adds a penalty to the loss function equal to the sum of the absolute values of the model coefficients, while Ridge regression (L2 regularization) applies a penalty proportional to the square of the coefficients. Both approaches aim to minimize error on the training data while constraining model weights.

In elastic net, the mixing parameter alpha (α) determines the balance between L1 and L2 regularization (larger *α* gives relatively more L1; smaller α gives relatively more L2). The lambda (*λ*) parameter is a tunable hyperparameter that controls the overall penalty strength—higher values of *λ* result in stronger penalization. In this study, *α* was set to 0.5, and *λ* was optimized using k-fold cross-validation from the *glmnet* package:

$$\begin{array}{c}Model\:=\:cv.glmnet(X,\;y,\;alpha\:=\:0.5,\;nfolds\:=\:10)\\Model\$lambda.1se\end{array}\\$$

At the second stage of predictor selection for regression modeling, *partial least squares (PLS)* regression was used to assess variable importance [35]. This method is particularly effective when the dataset contains highly correlated predictor variables. PLS constructs new predictors—referred to as components—as linear combinations of the original variables. While it incorporates elements of principal component analysis, it additionally identifies a subset of latent variables within a transformed space where the relationship between the response variable and the predictors is maximized. This allows for the identification of the most influential variables, i.e., those most strongly correlated with the latent components.

$$model\:=\:train(BMI\:\sim\ .,data\:=\:BMI_{data},method\:=\:"pls")$$

Following variable selection using both elastic net and PLS regression, the resulting set of predictors was analyzed using *stepwise linear regression*. Although this method also performs variable selection, it is sensitive to multicollinearity and therefore requires prior dimensionality reduction. Stepwise selection was carried out using the step function from the stats package [36]:

$$\begin{array}{c}\begin{array}{c}\mathrm{model}\:\leftarrow\;\mathrm{lm}(\mathrm{BMI}\;\sim\;.,\;\mathrm{data}\:=\:{\mathrm{BMI}}_{\mathrm{data}})\\\mathrm{step}(\mathrm{model},\;\mathrm{direction}\:=\:"\mathrm{both}")\end{array}\end{array}$$

For model verification at this stage, model selection was also performed using cross-validation as follows:

$$\begin{array}{c}control\: <-\;trainControl(method\:=\:"cv",\;number\:=\:10)\\model\:<-\; train(BMI\sim.,data\:=\:BMI\_{data},\;method\:=\:"lmStepAIC",\;trControl\:=\:control)\end{array}$$

BMI predictions based on the final regression model were calculated using the *plsreg1* function from the *plsdepot* package with cross-validation [37]:

This layered analytical strategy—combining standardization, multiple model specifications, explicit time-series diagnostics, out-of-sample forecasting, bootstrap inference, and multi-method variable selection—was designed to ensure that the observed relationships between BMI and macrostructural predictors are both statistically robust and substantively meaningful.

## Results

At the initial stage of analysis, Pearson correlation coefficients were calculated to assess the strength of linear associations between BMI and each socio-economic indicator for 19-year-old males and females (Table 2).

**Table 2:** Pearson correlation coefficients between socio-economic indicators and BMI of 19-year-old males and females in Russia

Socio-economic indicators	**Males**			**Females**		
	*n*	*r*	*p*-values	*n*	*r*	*p*-values
*Average monthly per capita income (rubles)*	*19*	*0.97*	****	*19*	*0.52*	*ns*
*Gini index RF World Bank*	*20*	*0.32*	*ns*	*20*	*0.71*	*
*Gini index (Russia)*	*25*	*0.57*	*ns*	*25*	*0.64*	*ns*
**Urban population (absolute number)**	42	**0.57**	**	42	**0.74**	****
*Share of urban population %*	*42*	*0.74*	****	*42*	*0.87*	****
**Life expectancy at birth, total**	42	**0.39**	ns	42	**0.10**	ns
**Urban birth rate**	42	**−0.42**	ns	42	**−0.63**	***
*Urban fertility rate*	*42*	**−***0.42*	*ns*	*42*	**−***0.62*	***
**Death rate**	42	**0.59**	**	42	**0.79**	****
*Infant mortality rate*	*42*	**−***0.99*	****	*42*	**−***0.94*	****
**Protein supply (g protein per capita per day)**	42	**−0.17**	ns	42	**−0.42**	ns
**Daily caloric intake per person from animal protein**	42	**0.12**	ns	42	**−0.13**	ns
* Animal products: protein (g/day per capita)*	*42*	*0.12*	*ns*	*42*	**−***0.13*	*ns*
* Meat supply per capita (kg/year)*	*42*	*0.23*	*ns*	*42*	**−***0.03*	*ns*
**Total meat consumption in Russia**	42	**0.33**	ns	42	**0.06**	ns

Bold indicates variables included in the subsequent regression analysis

Italics indicate variables excluded from the regression analysis

*df* = n−2

*ns, non-significant: p> 0.05*

*n*—number of observations used to compute the correlation coefficient, *r*—Pearson correlation coefficient, *p*—significance level: *ns*: *p* > 0.05, *p ≤ 0.05, **p ≤ 0.01, ***p ≤ 0.001, ****p ≤ 0.0001

Among males, the strongest correlations were observed with infant mortality (*r* = −0.99) and average monthly per capita income (*r* = 0.97). These exceptionally high coefficients reflect highly similar patterns of variation over time, suggesting the influence of common underlying factors driving changes in both BMI and these socio-economic indicators. For females, a similarly strong negative correlation with infant mortality was observed (*r* = −0.94). Such high correlations may be partly attributable to the nature of the BMI data: in certain years, BMI values were estimated (approximated from available data) rather than directly measured. This may have reduced variability in the BMI data and, consequently, inflated correlation coefficients with other similarly trending indicators. These high coefficients should not be taken as evidence of genuine associations between the variables, as they likely reflect shared temporal trends rather than substantive relationships.

To prevent the identification of spurious relationships in the modeling process, variables showing excessively high correlations with BMI were excluded. Additionally, indicators with limited data availability were removed, as were redundant variables reflecting the same underlying phenomenon—for instance, *urban fertility rate* was excluded due to its strong overlap with *urban birth rate*. Following this variable selection process, 7 of the original 15 socio-economic indicators were retained for further analysis (Table 2).

The intercorrelations among socio-economic indicators are presented in Table S1 and reveal substantial overlaps between several variables, necessitating variable selection to reduce redundancy and avoid multicollinearity.

Variable selection was carried out using two complementary methods. In the elastic net regression for males, the optimal lambda (*λ*) value (lambda.1se) was 0.008, with *α* set at 0.5. Six predictors were assigned nonzero coefficients: urban population in Russia (−0.05), life expectancy at birth (0.49), urban birth rate (−0.01), death rate (0.52), protein supply (−0.01), and meat consumption in Russia (0.06) (Table 3).

**Table 3:** Regression coefficients obtained using elastic net regression

		**Males**	**Females**
(Intercept)		25.16	26.54
Urban population (absolute number)		−0.05	0.05
Life expectancy at birth		0.50	0.11
Urban birth rate		−0.01	
Death rate		0.52	0.20
Protein supply (g protein per capita per day)		−0.02	
Daily caloric intake per person from animal protein			0.03
Total meat consumption in Russia		0.07	
1se	Lambda	0.0067	0.0124
	Measure	0.0057	0.0039
	SE	0.0012	8e-04

For females, elastic net regression yielded an optimal lambda of 0.013. Four predictors had nonzero coefficients: urban population (0.05), life expectancy at birth (0.11), death rate (0.20), and daily caloric intake (0.03). As in the male model, the most influential predictors were death rate and life expectancy at birth.

**Fig. 1:**
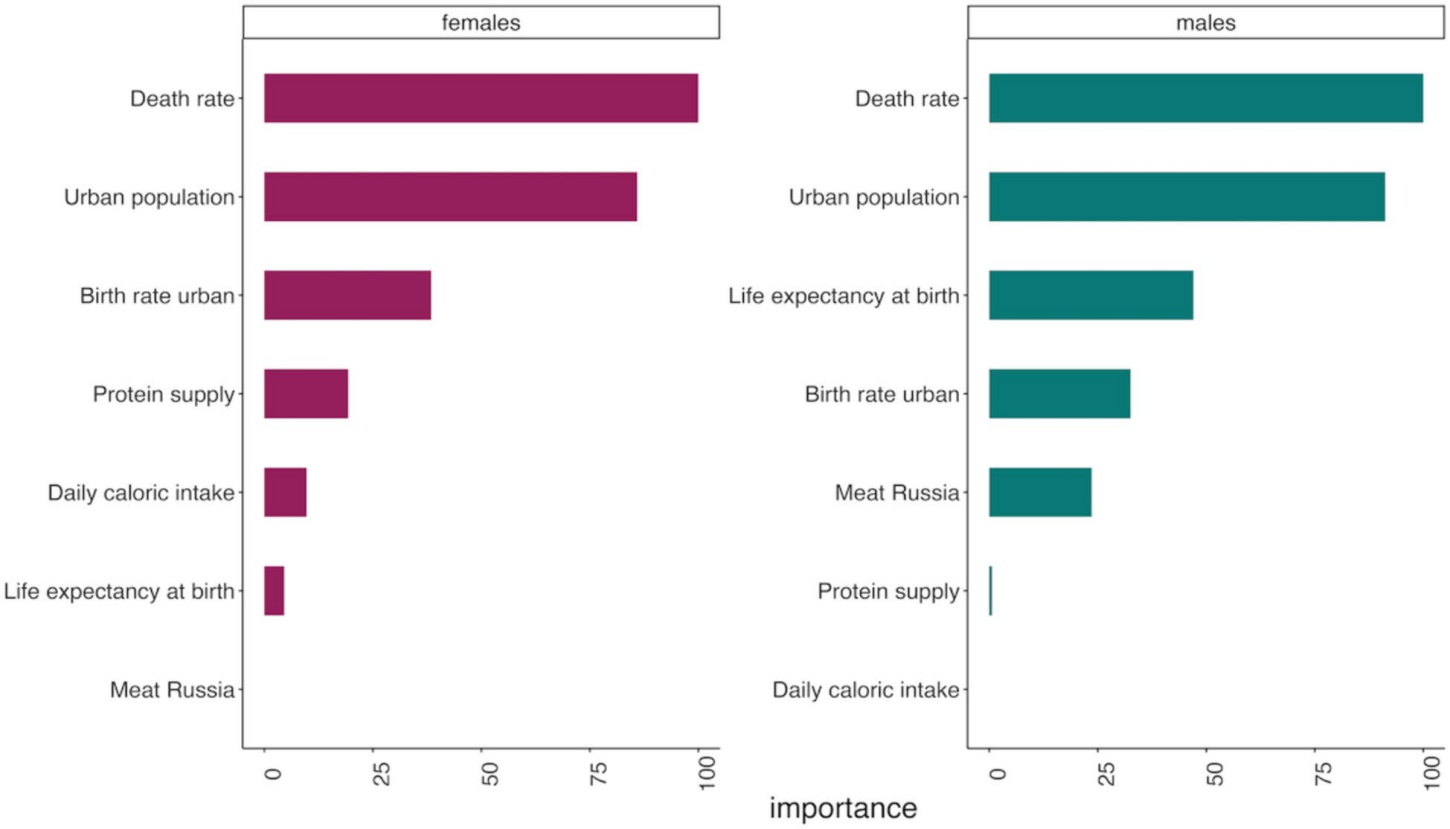
Importance of socio-economic indicators in partial least squares regression models predicting BMI among 19-year-old males and females in Russia

As a second selection criterion, variable importance was evaluated using partial least squares (PLS) regression, which confirmed the relatively low contribution of the nutrition-related variable *protein supply*, leading to its exclusion from the final model (Fig. 1). We selected PLS regression as a dimensionality reduction technique because unlike PCA, it explicitly accounts for the response variable when constructing latent components. This makes it more suitable for predictive modeling of BMI, as it captures shared variance between predictors and the outcome, rather than merely maximizing variance among predictors.

For females, PLS regression was used to evaluate variable importance, leading to the exclusion of total meat consumption in Russia, as it contributed minimally to the model.

Table 4 presents the results of the PLS analysis, showing how the predictors load on the latent components along with the relevant model diagnostics.

**Table 4:** Results from PLS regression analysis: predictor loadings on PLS components, regression coefficients, and cross-validated performance metrics (males and females)

Features	**Males**			**Females**		
**Loadings**	Comp 1	Comp 2	Comp 3	Comp 1	Comp 2	Comp 3
Urban population	**0.69**	0.01	−**0.76**	**0.69**	0.01	−**0.76**
Life expectancy at birth, total	−0.02	**0.44**	−0.06	−0.02	**0.44**	−0.06
Urban birth rate	−**0.61**	0.32	−0.13	−**0.61**	0.32	−0.13
Death rate	**0.60**	−0.25	**0.69**	**0.60**	−0.25	**0.69**
Protein supply (g protein per capita per day)	−**0.45**	**0.46**	−0.05	−**0.45**	**0.46**	−0.05
Daily caloric intake per person from animal protein	−0.22	**0.52**	−0.01	−0.22	**0.52**	−0.01
Total meat consumption in Russia	−0.01	**0.50**	−0.06	−0.01	**0.50**	−0.06
SS loadings	1.45	1.09	1.08	1.28	1.05	1.10
Proportion Var	0.21	0.16	0.15	0.18	0.15	0.16
Cumulative Var	0.21	0.36	0.52	0.18	0.33	0.49
**Coefficients**	Outcome			Outcome		
Urban population	0.16	0.18	−0.09	0.05	0.09	0.03
Life expectancy at birth, total	0.09	0.17	0.32	0.01	0.05	0.07
Urban birth rate	−0.12	−0.11	−0.14	−0.05	−0.06	−0.04
Death rate	0.17	0.20	0.49	0.06	0.09	0.21
Protein supply (g protein per capita per day)	−0.05	−0.01	−0.03	−0.03	−0.02	0.00
Daily caloric intake per person from animal protein	0.03	0.10	0.13	−0.01	0.03	0.07
Total meat consumption in Russia	0.08	0.16	0.17	0.00	0.05	0.03
**No. of comp**	**RMSE**	**R-squared**	**MAE**	**RMSE**	**R-squared**	**MAE**
1	0.34	0.51	0.28	0.15	0.59	0.13
2	0.26	0.74	0.21	0.10	0.82	0.08
3	0.13	0.92	0.10	0.06	0.93	0.05
**No. of comp**	**RMSESD**	**RsquaredSD**	**MAESD**	**RMSESD**	**RsquaredSD**	**MAESD**
1	0.08	0.19	0.06	0.04	0.20	0.03
2	0.04	0.11	0.03	0.02	0.09	0.02
3	0.04	0.05	0.03	0.02	0.05	0.01

All loadings with an absolute value ≥ 0.40 are highlighted in bold to aid interpretation; this formatting has no effect on model estimation

To give the PLS solution substantive meaning, we interpret each component by the variables with the largest absolute loadings; these descriptive labels are offered purely for exposition and did not influence model estimation or selection. The first component reflects a pattern of urban concentration under demographic stress, combining higher urban population and elevated death rates with lower urban birth rates. The second component aligns improvements in living conditions with nutrition, linking greater life expectancy to stronger proxies of dietary availability and quality (such as animal-based calories, protein, and meat) alongside a lower death rate. By contrast, the third component isolates short-run mortality spikes that are only weakly related to contemporaneous urban structure, capturing a residual “crisis” dimension against a slower-moving backdrop of urban change.

Across sexes, these components explain a substantial share of predictor variance and improve predictive performance as components are added (see Table 4 diagnostics). The key predictors identified in the PLS analysis—especially urbanization, mortality, and life expectancy—also demonstrated stable signs and significance across elastic net and stepwise regression models, supporting their robustness across estimation techniques. The prominence of life expectancy and death rate within components 1–2 aligns with their large, statistically significant coefficients in the final stepwise models, reinforcing their role as core predictors of BMI dynamics.

Based on the most informative predictors identified in the previous steps, stepwise linear regression was performed (Table 5). In the male model, *urban birth rate* was excluded, while all remaining variables had regression coefficients significantly different from zero. In the female model, all four selected predictors yielded statistically significant coefficients and were retained in the final regression equation.

**Table 5:** Stepwise linear regression results for males and females

**Males**	*β*-coeff.	*CI* 2.5%	*CI* 97.5%	*SE*	*t*-value	Pr (>|t|)	VIF	**Females**	*β*-coeff	*CI* 2.5%	*CI* 97.5%	*SE*	*t*-value	Pr (>|t|)	VIF
Intercept	25.16	25.14	25.19	0.01	2281.04	< 2 × 10^−16^***		Intercept	26.55	26.53	26.57	0.01	3251.94	< 2 × 10^−16^***	
Urban population (absolute)	−0.06	−0.09	−0.03	0.01	−3.99	0.00029***	1.93	Urban population (absolute)	0.04	0.02	0.06	0.01	3.59	0.00096***	2.06
Life expectancy at birth, total	0.57	0.54	0.61	0.02	34.94	< 2 × 10^−16^***	1.64	Life expectancy at birth, total	0.12	0.08	0.16	0.02	6.09	4.8 × 10^−^^7^***	4.47
Death rate	0.56	0.53	0.60	0.02	33.13	< 2 × 10^−16^***	2.56	Death rate	0.23	0.20	0.26	0.01	18.09	< 2 × 10^−16^***	2.68
								Daily caloric intake	0.04	0.01	0.08	0.02	2.54	0.0155*	4.79
**RSE**	0.07	38 DF						**RSE**	0.05			37 DF			
**Multiple *R***^**2**^	0.98	**Adj.R**^**2**^	0.98					**Multiple *R***^**2**^	0.96			**Adj.*R***^**2**^	0.95		
**F-statistic**	710.8	< 2.2 × 10^−16^						**F-statistic**	200.60			< 2.2 × 10^−16^			
**Durbin-Watson test**	1.07	4.4 × 10^−^^5^						**Durbin-Watson test**	0.751	3.9×10^−8^					
**Nonconstant variance score test**	Chi-square = 4.47		*p* = 0.03					**Nonconstant variance score test**	Chi-square = 1.35		*p* = 0.25				
**Shapiro–Wilk normality test residuals**	*W* = 0.94		*p*-value = 0.02					**Shapiro–Wilk normality test residuals**	*W* = 0.93		*p*-value = 0.01				

*p*, significance level; *ns*, *p* > 0.05, *p ≤ 0.05, *p* ≤ 0.01, ***p ≤ 0.001

**Fig. 2:**
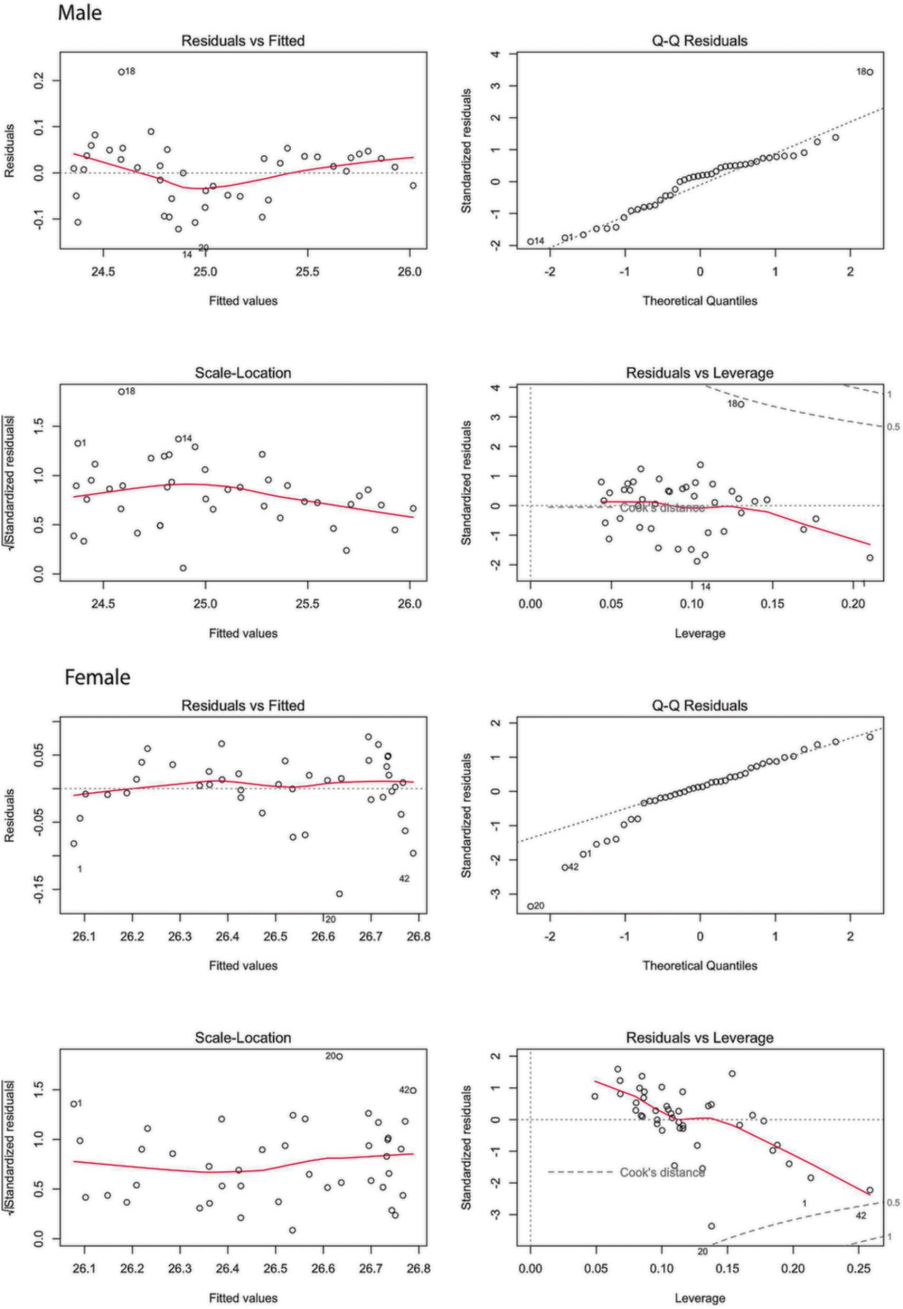
Diagnostic plots for the final regression model

Following stepwise regression, all retained coefficients had variance inflation factors (VIFs) between 1 and 5, indicating moderate multicollinearity, suggesting some correlation among predictors, but not to a problematic extent. The Durbin–Watson test indicated autocorrelation, which is consistent with the presence of time trends in the input data. Stationary residuals were achieved only in the model for females, while the assumption of normally distributed residuals was violated in both models. Diagnostic plots were generated to visualize model performance and residual behavior (Fig. 2).

As a result of the multistage statistical analysis, final regression equations were developed, representing theoretical models of BMI dynamics in response to temporal changes in external socio-economic and nutritional indicators:

- *Equation (1)*: BMI (males) = 25.16−0.06 × urban population of Russia + 0.57 × life expectancy at birth + 0.56 × death rate
- *Equation (2)*: BMI (females) = 26.55 + 0.04 × urban population of Russia + 0.12 × life expectancy at birth + 0.23 × death rate + 0.04 × daily caloric intake

The final regression models developed for males and females, based on temporal variation in socio-economic factors, revealed that demographic indicators (life expectancy and mortality) show large associations in level models but attenuate under smooth-trend controls; urbanization measures remain the most stable correlates across specifications (Table 5). Urbanization indicators—especially the share of the urban population—were the most stable correlates of BMI; life expectancy and mortality attenuate once smooth time structure is accounted for. Patterns differed by sex: male urbanization is robustly negative; female associations are small and model sensitive.

Because the original series are nonstationary, we tested multiple ways of handling that non-stationarity to check the robustness of our results. At levels, the augmented Dickey–Fuller test confirmed unit roots in many variables. First-differencing improved stationarity for most of them—Δlife expectancy and Δmortality, for example, became stationary—whereas ΔBMI yielded mixed outcomes, reaching stationarity in one sex but not the other.

### Model A — First-difference specification (Δ)

For males, this model explains *R*^2^≈0.75 (*Adj-R*^2^≈0.66) with *DW*≈0.75 and residual ADF *p*≈0.058 (borderline significant). For females, *R*^2^≈0.69 (*Adj-R*^2^≈0.59), *DW*≈1.37, and residual ADF *p*≈0.75. The effects of Δlife expectancy and Δmortality weaken and become statistically nonsignificant, suggesting their level-wise associations are largely trend-driven, whereas Δurban birth rate (positive) and Δinfant mortality (negative) remain significant in at least one sex-specific model.

### Model B — Dynamic specification with a lagged dependent variable (ARDL [1,0])

Including BMIₜ₋₁ captures strong inertia and yields stationary residuals (ADF *p*≈2 × 10^−5^; *DW*≈1.60). As expected, explanatory power is high. Among males, contemporaneous life expectancy and mortality remain positively significant after accounting for inertia, while the urban-population share is negatively significant. Among females, life-expectancy and mortality effects weaken and become nonsignificant, and the urban-population share effect is small and model dependent (frequently negative but not uniformly significant).

### Model C—Smooth-trend specification

Adding a flexible time spline for calendar year produces stationary residuals (ADF *p* < 10^−⁶^) with DW≈1.71 for males and 2.01 for females. In this setting, life expectancy and mortality lose statistical significance for both sexes, indicating that a broad smooth trend dominates their signal. In contrast, urbanization indicators remain robust (urban population share negative; urban birth rate positive), while nutrition proxies show mixed and generally weaker effects.

Model diagnostics (Table S2) indicate that residual autocorrelation is largely controlled in the ARDL and spline specifications (Durbin–Watson≈1.6–2.0), and ADF tests confirm residual stationarity in nearly all cases (*p* < 0.001). The ARDL(1,0) models account for 95.4% of the variance in male BMI and 93.1% in female BMI, and their rolling one-step forecasts yield a mean absolute error below 0.01 BMI units—effectively negligible relative to year-to-year variation. These forecast errors are conditional on the future path of exogenous variables; longer-horizon projections should be interpreted as scenario based rather than unconditional predictions.

Across all three specifications, conclusions are consistent: (i) differencing suppresses trend-induced correlations and attenuates life-expectancy and mortality effects, (ii) the dynamic model shows that demographic proxies remain linked to male BMI after accounting for inertia, (iii) the smooth-trend model absorbs most of the life-expectancy and mortality signal, while urbanization indicators persist as the most stable correlates. These findings justify cautious interpretation and joint reporting of DW and ADF statistics alongside coefficients, and they confirm the informational value of several key predictors for subsequent analysis.

**Fig. 3:**
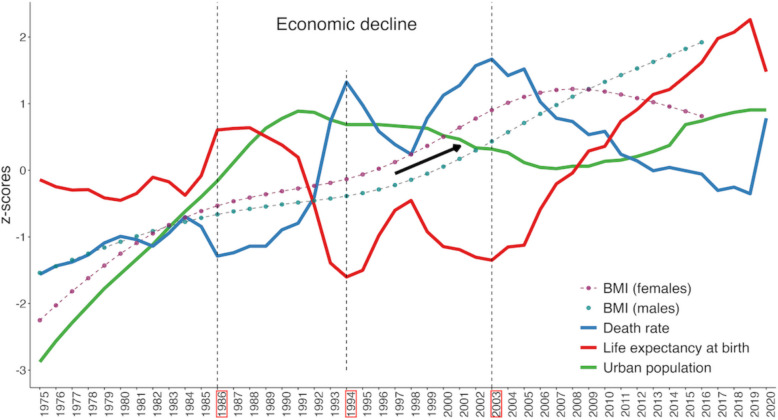
Temporal dynamics of BMI in the context of long-term changes in key demographic indicators. Note: Annual BMI estimates are taken directly from the NCD-RisC dataset without additional smoothing or interpolation

Figure 3 presents a visual summary of the joint temporal variation in the indicators studied. The data clearly show that life expectancy and mortality rate exhibit opposing (mirror-like) trajectories over time, changing in parallel but in opposite directions. Increases in life expectancy coupled with declines in mortality reflect improvements in living conditions at the national or regional level. From 1975, the starting point of this study, until the mid-1980s, a gradual increase in BMI was observed in both sexes, occurring alongside relatively minor fluctuations in life expectancy and mortality. Beginning in the mid-1980s, life expectancy declined sharply, reaching its lowest point in the mid-1990s, while mortality rose significantly during the same period (1986–1994). During this time, the increase in BMI plateaued, with the steady upward trend observed earlier slowing considerably. In the late 1990 s (1994–1998), as life expectancy began to recover and mortality rates declined, BMI values in both males and females resumed their upward trajectory—a trend that continued over an extended period thereafter.

Starting in the early 2000s—specifically in 2003—a sustained increase in life expectancy is observed, reflecting an overall improvement in socio-economic conditions. This trend continues until the onset of the COVID-19 pandemic and is accompanied by a continued rise in average BMI values among both males and females. Among females, however, the increase in BMI levels plateaus around 2007–2008, followed by a slight decline through the end of the observation period (2016). Throughout this period, mortality rates consistently moved in the opposite direction, declining as life expectancy increased, reinforcing the interpretation of improving living conditions.

A somewhat different pattern is observed for the share of the urban population. A sharp increase in urbanization is evident up to the early 1990 s, which closely corresponds with the upward trend in BMI for both sexes during that period. From the early 1990 s to approximately 2006–2007, the proportion of the urban population declines slightly, followed by a modest increase that continues through the end of the study period. The trajectory of BMI, however, does not fully mirror these changes. Among males, BMI continues to rise steadily through 2016, despite the temporary decline in urban population share in the early 2000s. Among females, BMI increases until 2006–2007 and then shows a slight decrease toward the end of the period.

The results suggest that long-term changes in the selected socio-economic indicators are associated with temporal trends in BMI. The patterns of variation among the predictors included in the model do not demonstrate a strictly linear relationship with BMI, which underscores the plausibility and nuanced nature of the identified associations.

Using the developed regression models and long-term projections of socio-economic trends in Russia, estimated BMI values were calculated for young adult males and females for the near future—specifically the year 2050. Due to the lack of available forecasts for nutritional factors and caloric intake and considering that these variables had the lowest importance in the models, they were excluded from the prediction.

**Fig. 4:**
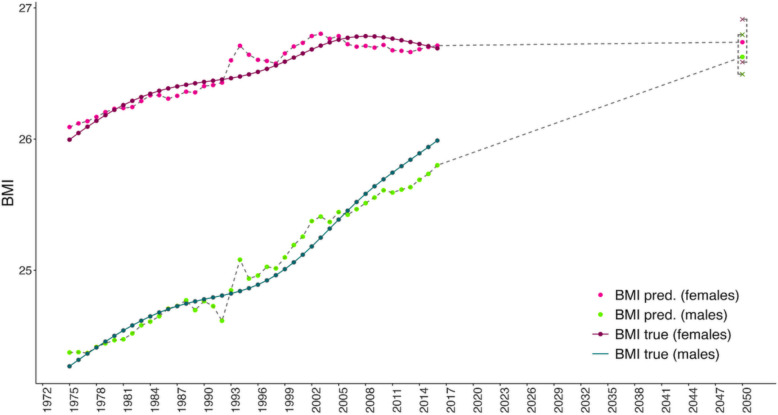
Observed and predicted BMI trajectories for Russian males and females, 1975–2050. Notes: Solid lines represent annual BMI estimates from NCD-RisC; dashed lines are model projections (teal for males, magenta for females). Dashed lines show BMI values predicted by the stepwise linear regression model. Crosses indicate 95% confidence intervals for predicted BMI values in 2050, derived from the standard errors of model residuals. These intervals reflect forecast uncertainty based on in-sample model fit

According to socio-economic projections, by 2045–2050, the urban population in Russia is expected to reach 110.6 million, life expectancy is projected to rise to 79.83 years, and the mortality rate is expected to decline to 9.9 [38, 39]. Based on the previously developed regression equations (theoretical models), an approximate forecast of BMI dynamics among Russian males and females was made for the year 2050 (Fig. 4). Under current socio-economic scenarios, the BMI of males is projected to increase to 26.62 ± 0.1 (*CI* 26.5–26.8) (from 25.9 in 2016), while for females a more modest increase is expected—from 26.7 in 2016 to 26.74 ± 0.1 (*CI* 26.6–26.9) in 2050 (Fig. 4).

The developed models were validated using an alternative statistical approach, based on the same set of socio-economic indicators. This validation was carried out using partial least squares (PLS) regression (plsreg1 function), through which a separate regression equation was generated to calculate predicted BMI values. Compared to the stepwise linear regression, this method produced less robust predictive results, with an *R*^2^ of 0.68. According to the PLS model, the projected average BMI values for males and females in 2050 are nearly identical: 26.978 for males and 26.973 for females. These predictions closely align with those obtained from the stepwise linear regression model, both indicating an increase in BMI by 2050. In both cases, the BMI of males is projected to increase more than that of females.

Overall, the use of an alternative statistical algorithm confirmed the robustness and consistency of the identified associations between temporal changes in selected socio-economic indicators and BMI dynamics.

**Fig. 5:**
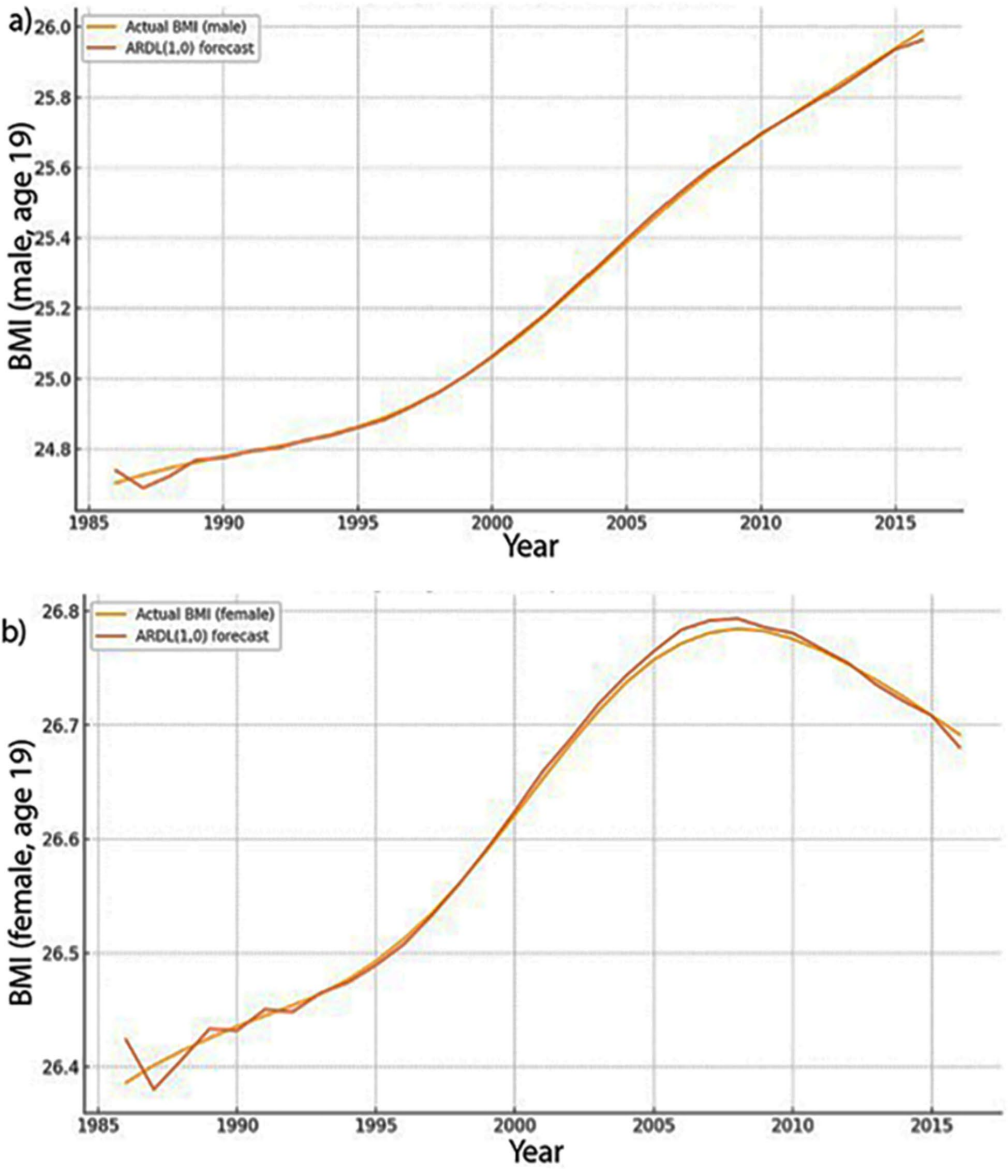
One-step-ahead rolling-origin forecasts (ARDL (1,0)) vs. observed BMI at age 19: **a** males. **b** Females. Russia, 1985–2016

To evaluate predictive performance, we implemented a *rolling-origin (walk-forward)* validation procedure. At each step, the ARDL(1,0) model was re-estimated on an expanding historical window and used to produce a one-step-ahead BMI forecast. Forecasts used only information available at time t: the lagged dependent variable and, for contemporaneous predictors, simple nowcasts (random-walk or AR(1)) generated from data up to t. Accuracy was assessed with mean absolute error (MAE) and root-mean-squared error (RMSE) and benchmarked against two baselines: (i) A naïve random-walk model (ŷ_{t + 1} = y_t) and (ii) a trend-only specification with linear and quadratic terms in calendar year. Across both sexes, the ARDL(1,0) model substantially outperformed the baselines. For males, MAE/RMSE were 0.0084/0.0124 versus 0.0423/0.0464 (random walk) and 0.0564/0.0749 (trend only). For females, MAE/RMSE were 0.0065/0.0096, again much lower than the baselines (Table S4, Fig. 5).

The *resampling analysis* broadly confirmed the ARDL coefficients (Table S5). In males, every draw retained a positive life-expectancy effect (mean = 0.022, 95% *CI* 0.011–0.037) and a negative urban-population effect (mean = −0.019, 95% *CI* −0.035 to −0.008), with inclusion frequencies of 100% and 98%, respectively. Mortality rate was also positive and precise (CI excludes 0 in 94% of runs), whereas the urban birth-rate coefficient crossed zero and was kept in only 88% of samples, indicating weaker evidence. Infant mortality remained negatively associated (*CI* −0.029 to −0.003, 91% inclusion) but with a small effect size. For females, the urban-population share again emerged as the most reliable predictor (mean = −0.021, 95% *CI* −0.038 to −0.009, 100% inclusion), followed by a modest positive life-expectancy effect (95% *CI* 0.004–0.029). Mortality and urban birth-rate coefficients displayed wide intervals that straddled zero, underscoring their fragility. Overall, the bootstrap highlights two robust patterns: (i) strong BMI inertia (see Table S4) and (ii) a reliable negative link between urbanization and BMI in males, with a smaller, specification-sensitive association in females, while other demographic effects—especially in females—appear more sensitive to model assumptions and sampling variability.

These results demonstrate that including BMI inertia (lagged BMI) and contemporaneous structural predictors markedly improves short-term forecast accuracy over trend-only and naïve extrapolations (Table S4; Fig. 5). Forecasts use only information available at time t (lagged predictors/nowcasts) and are conditional on assumed exogenous trajectories; longer-horizon projections are scenario based rather than unconditional.

## Discussion

This study provides new insights into the long-term dynamics of BMI among 19-year-old males and females in Russia, examined in the context of demographic and socio-economic change over four decades. By combining differencing, dynamic modeling, and trend-adjusted specifications, we were able to separate the temporal structure of the data from potentially substantive associations. This approach helped to distinguish variables that merely covary with BMI due to shared time trends from those that retain stable associations after accounting for temporal dependence.

Across all modeling specifications, urbanization indicators—particularly the share of the urban population—emerged as consistent and statistically robust predictors of BMI variation. From an ecological urbanization-health perspective, the urban context can influence BMI through three linked channels: the built environment (e.g., walkability, transport, land use), access to services (food retail, healthcare, recreation), and behavioral patterns (occupational activity, commuting, diet). These mechanisms provide a plausible pathway by which urbanization co-moves with BMI in our national series. While our design is ecological and cannot test these pathways, the pattern is consistent with this framework. However, while the association was stable across models, the corresponding effect sizes were small, indicating that urbanization exerts a modest influence on BMI levels rather than a large one. In contrast, life expectancy and mortality, although initially strong predictors, attenuated or disappeared in models that adjusted for temporal trends, suggesting that their apparent association with BMI may be partly endogenous to broader developmental processes.

Sex-specific differences were also pronounced. Among males, life expectancy and mortality retained positive associations with BMI in several models, and the association with urbanization remained negative; among females, these effects weakened or disappeared. The BMI trajectory for females plateaued or declined in later years, possibly reflecting distinct behavioral or biological responses to environmental change, echoing patterns observed in cross-national research. We treat these sex-specific explanations as hypotheses consistent with prior literature rather than confirmed findings; because our design is ecological and based on national time series, it cannot adjudicate individual-level mechanisms.

Methodologically, the use of multiple time-series strategies—including first-difference models, ARDL(1,0) specifications, and smooth-trend models—strengthened the robustness of our conclusions. The superior forecasting performance of the ARDL(1,0) model, together with the consistent urbanization effects across specifications, affirms the stability and interpretive value of the identified associations. However, forecast implications should be read cautiously: our forecasts use only information available at time t (lagged predictors/nowcasts), and longer-horizon projections are conditional on assumed paths of exogenous variables. Long-horizon BMI trajectories are not forecasts of political change; projections are conditional on exogenous paths and cannot anticipate policy or geopolitical shocks.

This analytical rigor is particularly important given the broader scientific context, where body composition trends have proven highly variable across populations and historical periods.

The variability of secular trends in total body size (height and weight) across different populations and historical periods presents a significant challenge in identifying the key drivers and underlying causes of long-term changes in body composition. This phenomenon appears to result from the interaction of endogenous (e.g., genetic, epigenetic) and exogenous (e.g., ecological, climatic, socio-economic, sociocultural) influences. Over the past century, however, improvements in living conditions have increasingly been recognized as the dominant force shaping body composition trends [40–44].

The relevance of the present study lies in its attempt to assess the differentiated contributions of various socio-economic indicators to secular changes in BMI, a widely used proxy for body composition, and, more broadly, to long-term transformations in the somatic status of modern populations during the second half of the twentieth and early twenty-first centuries.

Although the observed patterns reveal similarities in variability across indicators, they do not necessarily imply causal relationships. Nonetheless, the findings point to shared trends over time. Previous studies on BMI variation have shown that only a small proportion of its variability is directly attributable to socio-economic processes [45]. However, this may still reflect the influence of overarching factors that link social and economic development to changes in physical status. For example, as living standards and overall well-being improve, both socio-economic indicators and physical health measures tend to rise concurrently, with BMI serving as one expression of such broader shifts.

The relationship between secular trends in body composition and the socio-economic and demographic indicators examined in this study may not result from their direct influence but rather be mediated by other factors. For instance, improvements in nutritional conditions or easier access to food choices may accompany socio-economic changes, and it is these intermediate shifts that ultimately contribute to changes in somatic traits, most notably BMI. In this sense, temporal variation in socio-economic parameters may simply mirror changes in somatic indicators over time, without indicating a direct causal pathway.

An analysis of BMI variability over the past 40 years reveals a notably unstable trajectory. BMI increased steadily until the mid-1980s, plateaued through the mid-1990s, and then resumed an upward trend in both sexes. Among males, this rise has continued to the present day, while among females, average BMI values have slightly declined over the past decade.

This pattern may be linked to broader external influences, particularly the well-documented and prolonged decline in quality of life in Russia from the mid-1980s to the early 2000s. The period was marked by a profound economic transition and political upheaval, beginning with the reforms of perestroika and glasnost and culminating in the dissolution of the Soviet Union in 1991. These events triggered a severe and multifaceted sociopolitical crisis, leading to widespread institutional collapse, the disintegration of state-supported healthcare and social welfare systems, and a sharp contraction in real wages. The population experienced sudden exposure to market-driven economic forces, which brought about rising unemployment, income inequality, food insecurity, and restricted access to medical services. This systemic destabilization contributed to a dramatic increase in mortality and a significant drop in life expectancy during the 1990s. The nutritional landscape also shifted: traditional food supply chains were disrupted, and many households experienced caloric insufficiency or adopted cheaper, lower-quality diets. Taken together, these factors likely exerted substantial biological stress on the population, contributing to the observed stagnation and fluctuations in BMI during this period.

One possible explanation for the recent decrease in average BMI among young females is the influence of sociocultural factors—specifically dominant aesthetic ideals related to body image. Throughout the twentieth century and into the present, Western cultures have promoted a thin ideal characterized by a slender physique with minimal body fat [46, 47]. In the twenty-first century, this has been complemented by the rise of the athletic or “fit” ideal, which emphasizes muscularity alongside leanness [47–50]. Despite this shift, both ideals share an emphasis on low body fat, indicating that the cultural preoccupation with thinness remains strong. Some studies have suggested that sex-specific differences in the variability of BMI may be linked to distinct genetic factors influencing this trait in males and females [51]. In China, for example, a more pronounced increase in male BMI has been observed in recent decades. This trend has been attributed to external factors that disproportionately affect males, such as higher alcohol consumption and more frequent eating outside the home [52]. However, other studies report that obesity is more prevalent among females, while males more often fall into the overweight (but not obese) category [53]. This observation does not fully align with the aforementioned hypotheses, and the cited study does not offer a clear explanation for this discrepancy.

Young females internalize these sociocultural standards through socialization and often engage in efforts to modify their appearance, body composition, or somatic characteristics to conform to socially accepted ideals of beauty and bodily aesthetics.

An analysis of linear relationships revealed extremely strong—almost absolute—associations between certain socio-economic indicators and body composition. For instance, variables such as infant mortality and average monthly per capita income exhibited correlation coefficients with BMI approaching unity. In biological research, correlations of this magnitude typically suggest a functional relationship between variables. In the present context, however, such high correlations may reflect the use of approximated rather than directly measured BMI values in some of the literature sources. As a result, including these indicators in the current modeling framework would be methodologically inappropriate, and they were therefore excluded from the analysis.

Among the remaining variables, the strongest associations with BMI were observed for urban population size, life expectancy, and mortality rate; for females, average daily caloric intake from animal protein also showed a meaningful relationship with BMI. However, while life expectancy and mortality appear strong in levels, their effects attenuate once temporal trends are controlled, whereas urbanization remains the most consistent correlate across specifications. Life expectancy and mortality rate are both broadly indicative of population health status, which is also reflected in BMI. Interestingly, both variables demonstrated positive correlations with BMI. At lower BMI levels, increases in BMI are generally associated with improved health outcomes, including reduced mortality and increased life expectancy. However, at higher BMI levels—indicative of excessive fat accumulation or obesity, the trend may reverse, with elevated BMI corresponding to increased mortality and reduced life expectancy. Excessive BMI is a well-documented risk factor for a range of metabolic disorders and is associated with shorter life expectancy [54]. Conversely, a decline in BMI below normal values may indicate deteriorating living conditions, which may also be linked to increased mortality and reduced life expectancy [55].

The relatively weak associations between nutritional indicators and BMI may appear counterintuitive, as dietary intake is generally assumed to directly influence fat accumulation. However, this likely reflects limitations in the available proxies, which are based on supply side, protein-focused aggregates rather than total caloric intake, fats, sugars, ultra-processed foods, or energy balance. At the ecological, annual scale used here—and for 19 years old whose BMI reflects cumulative exposures—these proxies offer limited predictive power once urbanization, demographic composition, and shared temporal trends are controlled for. Thus, our findings do not imply that nutrition has little effect on BMI but rather that the indicators used do not fully capture relevant dietary influences.

Based on the findings of this study, increases in indicators related to life expectancy are associated with rising BMI values in the Russian population. However, the rate and direction of BMI change differ between males and females. Previous research has highlighted the complexity of the relationship between BMI and life expectancy, with some studies reporting that higher BMI is associated with lower mortality risk [56]. These findings challenge the widely held view that higher BMI is consistently linked to increased mortality risk in adult populations.

The relationship between urban population size and BMI is less clear-cut. It may be hypothesized that urban areas are home to a greater proportion of individuals with higher socio-economic status and income, which could influence BMI levels. While some studies have found that urbanization is associated with increasing BMI [57, 58], others report an inverse relationship [59]. Although urbanization indicators consistently emerged as statistically robust predictors across model specifications, the strength of the association—measured by the size of the coefficients—was modest in both males and females. Moreover, the direction of the effect differed by sex, suggesting that the influence of urban population share on BMI may interact with other sex-specific or contextual factors. However, these differences were not formally tested for statistical significance, so at this stage they should be interpreted only as indicative trends. In our models, the urbanization association was negative for males and small and specification-sensitive for females.

According to the regression models, increases in the urban population were associated with a slight decrease in BMI among males and a slight increase among females. These divergent trends may reflect the largely random fluctuations in the urban population variable over the study period, as suggested by the very small coefficients observed in the regression equations. In recent decades, the urban population in Russia has remained relatively high and stable, and its variability may no longer meaningfully correspond to BMI trends. It is likely that stronger associations between urbanization and body composition were more pronounced in earlier historical periods, such as the first half of the twentieth century.

The relationship between living conditions and BMI has been documented in numerous studies. In high-income countries, an inverse relationship is often observed between BMI and socio-economic status, typically explained by differences in dietary habits and access to physical activity [60–62]. In the present study, the negative association between BMI and urban population levels among young males may be interpreted along similar lines.

One explanation for changes in BMI in response to lifestyle transformations is provided by the *nutrition in transition* model developed by Barry Popkin [63]. This model describes successive stages of human societal development in terms of shifts in dietary structure. At present, we are situated in the globalization stage, characterized by widespread availability of energy-dense foods and a departure from traditional diets. A defining feature of this stage is the transition from agrarian to industrial economies, which has led to declining physical activity across many occupations worldwide [63]. The findings of the present study are consistent with this framework.

Another model, developed to forecast the stabilization of BMI in the USA, suggests that increases in BMI—specifically the growing prevalence of obesity—are primarily a function of birthrate and the probability of being born into an obesogenic environment [64]. According to this model, other external factors have relatively limited influence on rising BMI, and irrespective of their trajectory, BMI is projected to reach a plateau in the USA by approximately 2030.

In several Asian countries, such as China, an increase in BMI over the past 50 years has been linked to socio-economic development and improved living standards [65]. In contrast, studies on secular trends in BMI in the USA have shown that in recent decades, the association between BMI and socio-economic indicators has weakened [66]. These findings are broadly consistent with the results of the current study. Some studies have questioned whether rising economic prosperity, improvements in healthcare, and better nutrition are the predominant external drivers of increases in body height. Instead, more compelling evidence has been found for associations with political freedom, hope for a better future, and expectations of social progress [67]. Given the direct relationship between height and BMI, it is reasonable to hypothesize that BMI may also, to some extent, be influenced by these broader societal processes.

Previous research on youth in Moscow has revealed a positive association between BMI and various socio-economic indicators, including GDP per capita, average monthly income, and the Gini index [26]. Across different global regions, changes in social and economic conditions are often accompanied by shifts in BMI, although the direction and strength of these associations vary by context. In some countries, BMI has increased in both males and females [9, 10]. In Finland, BMI has risen in males but declined in females, while in England and Scotland BMI has increased in females but remained stable in males [68, 69]. Design differences likely account for some divergent findings: our analysis uses ecological and national time-series data, whereas many comparators rely on individual-level cross-sectional or cohort designs; effect sizes are therefore not directly comparable. Together, these findings underscore the complexity and variability of BMI as an indicator and highlight its context-dependent associations, as also observed in the present study.

Recent scientific literature includes several pilot studies presenting theoretical models of BMI variability as a natural process, influenced by external factors. These studies suggest that the pattern of BMI change represents a balance between a natural physiological state and random fluctuations, driven by environmental influences. In other words, heavier individuals tend to become lighter over time, while lighter individuals tend to gain weight year over year [70]. This model may help explain the more modest BMI increase observed among females in the present study. In 2016, the average BMI for females was 26.7, which falls within the overweight category. A substantial increase beyond this level is unlikely, as it would imply a further deviation from conventionally defined normative values.

Based on the results of our analysis, and a review of previous studies, we can conclude that socio-economic indicators play a differentiated role in the long-term variability of BMI. However, it is important to note that their overall predictive power remains relatively limited [59]. As shown in the “Results” section, urbanization measures were the most persistent correlates across specifications, whereas life expectancy and mortality appeared strong in levels but attenuated after trend adjustments. Among all the factors examined, we therefore do not characterize life expectancy and mortality as “strongest predictors” in adjusted models; rather, they reflect shared long-run movement with BMI. Overall, our findings underscore the importance of explicitly addressing temporal structure when linking BMI trends to broader societal changes. By integrating time-series techniques into health and demographic research, future studies can better isolate enduring relationships from historical coincidence—and improve the reliability of long-term predictions under conditions of socio-economic transition.

Finally, we emphasize that the models presented here are designed to capture macro-level associations between socio-economic dynamics and BMI trends in the Russian population. They are not intended to infer individual-level causal relationships. All interpretations are accordingly framed at the population level, consistent with the ecological nature of the data. Recognizing this boundary helps avoid ecological fallacy and ensures that the findings are situated appropriately within the context of demographic and public health modeling.

## Limitations

This study has several important limitations. First, it relies on aggregate-level national time-series data, which are inherently nonstationary and autocorrelated. Although we applied differencing, ARDL, and smooth-trend adjustments, achieving full stationarity was not always possible, and, in some cases, detrending would have removed the long-run dynamics of interest.

Second, BMI values were drawn from smoothed estimates in international databases, limiting their short-term variability and potentially inflating correlations with similarly trended socio-economic series.

Third, the final regression models were estimated on a relatively small sample (*N*≈40 years) after multistage predictor selection. Even with cross-validation and alternative modeling approaches, the high in-sample *R*^2^ (> 0.95) raises the possibility of overfitting, and the coefficients should be interpreted with this in mind. Standard errors and *p*-values were not adjusted for post-selection inference. Reporting cross-validated *R*^2^ alongside in-sample fit would further mitigate this concern in future work.

Fourth, several potentially relevant covariates—such as education levels, healthcare access, and detailed dietary composition—were excluded due to incomplete historical data, introducing possible residual confounding. Additionally, the socio-economic predictors themselves are subject to historical measurement and estimation error, particularly in earlier decades.

Fifth, all relationships described are ecological and population level; they cannot be assumed to hold at the individual level. The models capture linear associations and do not test for potential nonlinear or threshold effects in BMI responses.

Finally, the projections are conditional scenarios that assume continuity of recent socio-economic trends. They cannot account for sudden policy, environmental, or demographic shocks (e.g., pandemics) and should be interpreted as illustrative rather than deterministic forecasts.

## Conclusions

Over four decades, BMI trends in 19-year-old Russians have evolved in parallel with broad demographic and socio-economic changes. After rigorous time-series adjustments, only a small subset of predictors proved consistently robust: urbanization indicators, particularly the share of the urban population, maintained statistically stable associations across specifications (negative in males; small and model sensitive in females), albeit with modest effect sizes. Demographic indicators such as life expectancy and mortality retained positive associations with male BMI in dynamic models but attenuated or disappeared under smooth-trend controls and were weak or absent in female models. National-level nutrition proxies contributed little explanatory power, likely due to their coarse resolution rather than true lack of influence.

Under a baseline scenario—assuming continuity of recent socio-economic patterns without major disruptions—the models project a modest BMI increase by 2050, reaching approximately 26.6 kg/m^2^ in males and 26.7 kg/m^2^ in females. These forecasts use only information available at time t (lagged predictors/nowcasts) and are conditional on the assumed trajectories of exogenous variables; they are scenario based rather than unconditional predictions.

Methodologically, this work demonstrates a transferable framework for analyzing short, nonstationary national time series by combining multiple model forms, robust variable selection, bootstrap inference, and genuine out-of-sample validation. Substantively, it highlights that in the Russian context, macrodemographic structure is a more consistent correlate of adolescent BMI trends than national-scale nutrition indicators.

Given the modest effect sizes for the most robust predictors, the findings emphasize the importance of distinguishing statistical stability from magnitude of association and of incorporating finer-grained behavioral, dietary, and environmental data in future studies. Such integration will be essential for clarifying causal pathways and improving the precision of long-term health forecasting.

## Supplementary Information

Supplementary Material 1: Table S1. Pearson correlation coefficients among socio-economic indicators in Russia. Table S2. Model Diagnostics Summary for BMI Regression Models. Table S3. Augmented Dickey–Fuller Test Results. Table S4. One-step-ahead rolling-origin forecast errors (31 test years, 1986–2016). Table S5. Bootstrap Summary.

## Data Availability

The data that support the findings of this study are available from the corresponding author (AM), upon reasonable request.
