# Supplementary material for: A theoretical model of secular body mass index dynamics among Russian adults under changing socio-economic conditions
Source: J Physiol Anthropol. 2025 Dec 29;44:34. doi: 10.1186/s40101-025-00415-5 (PMC12750930; doi:10.1186/s40101-025-00415-5)
Supplement: Supplementary file 1 — Supplementary Material 1: Table S1. Pearson correlation coefficients among socio-economic indicators in Russia. Table S2. Model Diagnostics Summary for BMI Regression Models. Table S3. Augmented Dickey–Fuller Test Results. Table S4. One-step-ahead rolling-origin forecast errors (31 test years, 1986–2016). Table S5. Bootstrap Summary. [file 40101_2025_415_MOESM1_ESM.doc]

**Table S1.** Pearson correlation coefficients among socio-economic indicators in Russia

|  | **GDPpc** | **UP** | **sUP** | **LEB** | **UBR** | **UFR** | **DR** | **IM** | **PS** | **DC** | **MS** | **MR** |
| --- | --- | --- | --- | --- | --- | --- | --- | --- | --- | --- | --- | --- |
| **GDPpc_Former** | 1,00 | 0,08 | 0,32 | 0,77 | 0,22 | 0,19 | 0,02 | -0,75 | 0,46 | 0,66 | 0,69 | 0,76 |
| **Urban population (absolute number)** | 0,08 | 1,00 | 0,96 | 0,00 | -0,67 | -0,53 | 0,60 | -0,61 | -0,51 | -0,25 | -0,03 | -0,01 |
| *Share of urban population %* | 0,32 | 0,96 | 1,00 | 0,15 | -0,59 | -0,48 | 0,62 | -0,78 | -0,39 | -0,10 | 0,10 | 0,14 |
| **Life expectancy at birth, total** | 0,77 | 0,00 | 0,15 | 1,00 | 0,52 | 0,58 | -0,50 | -0,36 | 0,71 | 0,85 | 0,85 | 0,85 |
| **Urban birth rate** | 0,22 | -0,67 | -0,59 | 0,52 | 1,00 | 0,97 | -0,87 | 0,45 | 0,91 | 0,76 | 0,62 | 0,55 |
| *Urban fertility rate* | 0,19 | -0,53 | -0,48 | 0,58 | 0,97 | 1,00 | -0,90 | 0,44 | 0,92 | 0,79 | 0,71 | 0,62 |
| **Death rate** | 0,02 | 0,60 | 0,62 | -0,50 | -0,87 | -0,90 | 1,00 | -0,62 | -0,81 | -0,64 | -0,53 | -0,45 |
| *Infant mortality rate* | -0,75 | -0,61 | -0,78 | -0,36 | 0,45 | 0,44 | -0,62 | 1,00 | 0,19 | -0,11 | -0,22 | -0,32 |
| **Protein supply (g protein per capita per day)** | 0,46 | -0,51 | -0,39 | 0,71 | 0,91 | 0,92 | -0,81 | 0,19 | 1,00 | 0,94 | 0,82 | 0,78 |
| **Daily caloric intake per person from animal protein** | 0,66 | -0,25 | -0,10 | 0,85 | 0,76 | 0,79 | -0,64 | -0,11 | 0,94 | 1,00 | 0,92 | 0,90 |
| *Animal products: protein (g/day per capita)* | 0,69 | -0,03 | 0,10 | 0,85 | 0,62 | 0,71 | -0,53 | -0,22 | 0,82 | 0,92 | 1,00 | 0,97 |
| *Meat supply per capita (kg/year)* | 0,76 | -0,01 | 0,14 | 0,85 | 0,55 | 0,62 | -0,45 | -0,32 | 0,78 | 0,90 | 0,97 | 1,00 |

**Table S2.** Model Diagnostics Summary for BMI Regression Models

| **Model specification** | **Sex** | **Durbin–Watson** | **ADF p-value (residuals)** | **R²** | **Adj. R²** | **RMSE** | **MAE** |
| --- | --- | --- | --- | --- | --- | --- | --- |
| **First-difference ΔBMIₜ ~ ΔXₜ** | Male | 0,75 | 0,06 | 0,75 | 0,66 | — | — |
|  | Female | 1,37 | 0,75 | 0,69 | 0,59 | — | — |
| **ARDL(1,0) BMIₜ ~ BMIₜ₋₁ + Xₜ** | Male | 1,60 | 2.15 × 10⁻⁵ | 0,95 | 0,95 | — | 0.008 |
|  | Female | 1,60 | 2.15 × 10⁻⁵ | 0,93 | 0,92 | — | 0.007 |
| **Spline trend BMIₜ ~ Xₜ + ns(year, df = 4)** | Male | 1,71 | 1.13 × 10⁻⁶ | — | — | — | — |
|  | Female | 2,01 | 9.39 × 10⁻⁹ | — | — | — | — |

**Table S3.** Augmented Dickey–Fuller Test Results

| **Variable** | **N** | **ADF Statistic (Level, Trend)** | **p-value (Level, Trend)** | **ADF Statistic (Diff, Drift)** | **p-value (Diff, Drift)** |
| --- | --- | --- | --- | --- | --- |
| **BMI_male** | 42 | -1.85 | 0.65 | -4.21 | 0.01 |
| **BMI_female** | 42 | -2.1 | 0.52 | -4.11 | 0.01 |
| **Life_expectancy** | 42 | -2.98 | 0.04 | -5.48 | 0.0 |
| **Mortality** | 42 | -1.6 | 0.78 | -4.59 | 0.0 |
| **Urban_population** | 42 | -0.74 | 0.88 | -5.92 | 0.0 |
| **Protein** | 42 | -1.25 | 0.74 | -4.97 | 0.0 |

**Table S4.** One-step-ahead rolling-origin forecast errors (31 test years, 1986–2016)

| **Model** | **Sex** | **MAE** | **RMSE** | **MAPE (%)** | **N Forecasts** |
| --- | --- | --- | --- | --- | --- |
| **Random walk** | Male | 0.0423 | 0.0464 | 0.1667 | 31 |
| **Trend-only (Year + Year²)** | Male | 0.0564 | 0.0749 | 0.2228 | 31 |
| **ARDL(1,0)** | Male | 0.0084 | 0.0124 | 0.0333 | 31 |
| **Random walk** | Female | 0.0164 | 0.0183 | 0.0618 | 31 |
| **Trend-only (Year + Year²)** | Female | 0.0446 | 0.0543 | 0.1674 | 31 |
| **ARDL(1,0)** | Female | 0.0065 | 0.0096 | 0.0246 | 31 |

*Notes:* MAE = mean absolute error; RMSE = root mean squared error; MAPE = mean absolute percentage error. ARDL(1,0) includes a lagged dependent variable and contemporaneous predictors; at forecast time t, contemporaneous predictors are nowcasted using information available up to t.

**Table S5.** Bootstrap Summary

| **Predictor** | **Sex** | **Mean Coefficient** | **95% CI Lower** | **95% CI Upper** | **Inclusion Frequency** |
| --- | --- | --- | --- | --- | --- |
| **Life Expectancy** | Male | 0.022 | 0.011 | 0.037 | 100% |
| **Urban Population Share** | Male | -0.019 | -0.035 | -0.008 | 98% |
| **Mortality Rate** | Male | 0.014 | 0.003 | 0.029 | 94% |
| **Urban Birth Rate** | Male | 0.012 | -0.005 | 0.026 | 88% |
| **Infant Mortality** | Male | -0.016 | -0.029 | -0.003 | 91% |
| **Life Expectancy** | Female | 0.017 | 0.004 | 0.029 | 95% |
| **Urban Population Share** | Female | -0.021 | -0.038 | -0.009 | 100% |
| **Mortality Rate** | Female | 0.01 | -0.002 | 0.024 | 89% |
| **Urban Birth Rate** | Female | 0.009 | -0.004 | 0.023 | 82% |
| **Infant Mortality** | Female | -0.013 | -0.025 | -0.002 | 93% |
